# Eight Mutations of Three Genes (*EDA*, *EDAR*, and *WNT10A*) Identified in Seven Hypohidrotic Ectodermal Dysplasia Patients

**DOI:** 10.3390/genes7090065

**Published:** 2016-09-19

**Authors:** Binghui Zeng, Xue Xiao, Sijie Li, Hui Lu, Jiaxuan Lu, Ling Zhu, Dongsheng Yu, Wei Zhao

**Affiliations:** Guanghua School of Stomatology, Hospital of Stomatology, Guangdong Provincial Key Laboratory of Stomatology, Sun Yat-sen University, Guangzhou 510055, China; zengbh@mail3.sysu.edu.cn (B.Z.); xiaoxue@mail.sysu.edu.cn (X.X.); lisijie@mail2.sysu.edu.cn (S.L.); luhui7@mail2.sysu.edu.cn (H.L.); lujxuan@mail.sysu.edu.cn (J.L.); zhulyxxx@163.com (L.Z.)

**Keywords:** hypohidrotic ectodermal dysplasia, *EDA*, *EDAR*, *EDARADD*, *WNT10A*

## Abstract

Hypohidrotic ectodermal dysplasia (HED) is characterized by abnormal development of the teeth, hair, and sweat glands. *Ectodysplasin A* (*EDA*), *Ectodysplasin A receptor* (*EDAR*), and *EDAR-associated death domain* (*EDARADD*) are candidate genes for HED, but the relationship between *WNT10A* and HED has not yet been validated. In this study, we included patients who presented at least two of the three ectodermal dysplasia features. The four genes were analyzed in seven HED patients by PCR and Sanger sequencing. Five *EDA* and one *EDAR* heterozygous mutations were identified in families 1–6. Two *WNT10A* heterozygous mutations were identified in family 7 as a compound heterozygote. c.662G>A (p.Gly221Asp) in *EDA* and c.354T>G (p.Tyr118*) in *WNT10A* are novel mutations. Bioinformatics analyses results confirmed the pathogenicity of the two novel mutations. In family 7, we also identified two single-nucleotide polymorphisms (SNPs) that were predicted to affect the splicing of *EDAR*. Analysis of the patient’s total RNA revealed normal splicing of *EDAR*. This ascertained that the compound heterozygous *WNT10A* mutations are the genetic defects that led to the onset of HED. Our data revealed the genetic basis of seven HED patients and expended the mutational spectrum. Interestingly, we confirmed *WNT10A* as a candidate gene of HED and we propose *WNT10A* to be tested in *EDA*-negative HED patients.

## 1. Introduction

Ectodermal dysplasia (ED) is defined as congenital alterations of at least two of ectodermal structures, such as teeth, hair, nails, and sweat glands [1,2]. ED is heterogeneous both clinically and genetically. There are 163 well-established clinical entrances in Group A of ED [3]. Seventy-seven genes have been proven to be the causative genes of 75 EDs [3]. Hypohidrotic ectodermal dysplasia (HED) is the most common form of ED [4]. HED is characterized by abnormal development of teeth (hypodontia or anodontia), hair (hypotrichosis), and sweat glands (hypohidrosis or anhidrosis) [5,6]. There are three subtypes of HED, namely: X-linked (XLHED, OMIM 305100), autosomal dominant (ADHED, OMIM 129490, or 614940), and autosomal recessive HED (ARHED, OMIM 224900, or 614941) [3]. *Ectodysplasin A* (*EDA*; Gene ID: 1896) is the candidate gene for XLHED [7]. The pathogenic genes of both ADHED and ARHED are *Ectodysplasin A receptor* (*EDAR*; Gene ID: 10913) and *EDAR-associated death domain* (*EDARADD*; Gene ID: 128178) [8,9].

There are three genes associated with HED that need to be confirmed. In 2012, Wisniewski and Trzeciak reported a patient with HED caused by c.252delG mutation in *EDA2R* [10]. In the same year, they reported another patient with HED caused by c.1074_1081del mutation in *TRAF6* [11]. In 2010, Cluzeau et al. reported 10 patients with HED caused by mutations in *WNT10A* [12]. *WNT10A* mutations accounted for 16% of ED patients in Cluzeau’s cohort [12].

In this study, we aimed to reveal the genetic basis of seven HED patients and test if *WNT10A* mutations contribute to the onset of HED in our patients. *EDA*, *EDAR*, *EDARADD*, and *WNT10A* genes were analyzed. We identified eight mutations, of which two are novel. We provided new evidence that *WNT10A* is a promising candidate gene of HED.

## 2. Materials and Methods

### 2.1. Subjects

This study consisted of seven unrelated HED families. Two families (families 6 and 7) had been tested for *EDA* mutation in our previous study, but no pathogenic mutation was found [5]. The other five families (families 1–5) were newly recruited ones. All of the patients were diagnosed as HED according to the criteria of having at least two of the three ectodermal abnormalities of teeth, hair, and sweat glands. Fifty healthy individuals were recruited as a normal control to rule out genetic polymorphism. This study was approved by the Ethical Review Committee of Guanghua School and Hospital of Stomatology, Sun Yat-sen University (ERC-[2013]-9, date of approval: 1 December 2013). Informed consent was obtained, and the Declaration of Helsinki was followed.

### 2.2. Mutation Detection of *EDA*, *EDAR*, *EDARADD*, and *WNT10A*

We collected about a 4-mL quantity of peripheral blood from each individual. Genomic DNA was extracted with QiaAmp Kit (Qiagen, Düsseldorf, Germany). Primers covering exons and flanking intronic sequences of *EDAR*, *EDARADD,* and *WNT10A* were designed using Oligo 6.0 (Molecular Biology Insights, Colorado Springs, CO, USA). These primer sequences were available in Tables S1–S3. The primer sequences of *EDA* were reported in previous work [5]. PCR was performed, and the products were sequenced with an ABI 3730XL genetic analyzer (Applied Biosystems, Foster City, CA, USA). The sequencing results were analyzed with Sequence Scanner Software v1.0 (Applied Biosystems, Foster City, CA, USA). Mutation nomenclature was used, with +1 corresponding to the A of the ATG translation initiation codon of the reference sequence NM_001399.4(*EDA*), NM_022336.3 (*EDAR*), and NM_025216.2 (*WNT10A*).

### 2.3. Splicing Analysis of *EDAR*

Total RNA was extracted from peripheral blood with HiPure Blood RNA Kits R4163 (Magen, Guangzhou, China). Reverse transcriptase-PCR (RT-PCR) was performed with 5 μg total RNA and M-MLV Reverse Transcriptase M1705 (Promega, Madison, WI, USA). Primers of 5′-CCATCGTCCTCATCATCATGTT-3′ (forward) and 5′-CACGTTGGCATACACATCGAG-3′ (reverse) were used to RT-PCR amplify *EDAR*.

### 2.4. Bioinformatics Study

Methods for undertaking a bioinformatics study were described in detail in our previous studies [13,14]. In brief, the amino acid sequence of human *EDA* (ENST00000374552) was compared with those of the mouse (ENSMUST00000113779), cattle (ENSBTAT00000016649), rhesus monkey (ENSMMUT00000024953), and chicken (ENSGALT00000007137) by using CLUSTAL X (1.81) [15]. The pathogenic effect of novel mutations was predicted by SIFT [16], Mutation Taster [17], PloyPhen2 [18], FATHMM [19], and PROVEAN [20,21].

### 2.5. Structural Modeling

We used the structure of protein Wnt-8 (PDB ID 4F0A; X-ray, resolution 3.25Å) as a homology model to perform structural modeling with Swiss PdbViewer v4.1 [22]. PyMol v1.5.0.3 (The PyMOL Molecular Graphics System, Version 1.8 Schrödinger, LLC.) was used to visualize the 3D structure of the wild-type Wnt-10a protein and mutated Wnt-10a protein.

## 3. Results

### 3.1. Clinical Report

**Figure 1:**
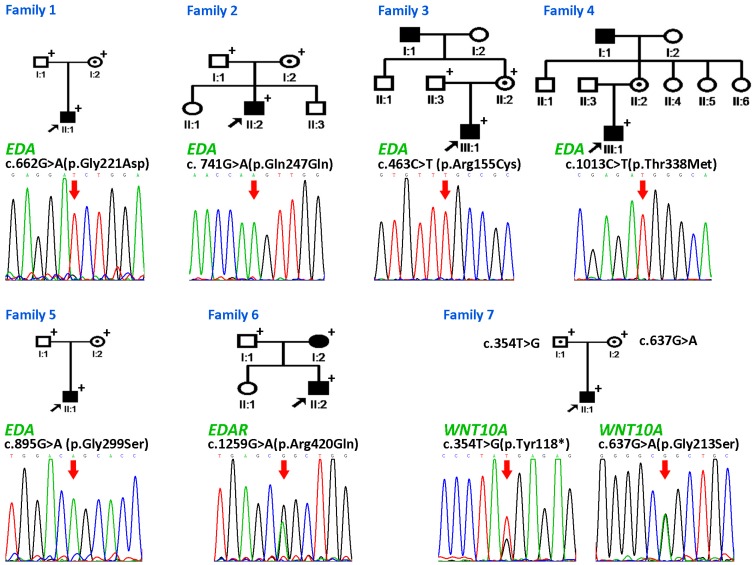
Pedigree and mutations of families 1–7. Families 1–5 harbored *Ectodysplasin A* (EDA) mutations: c.662G>A (p.Gly221Asp), c.741G>A (p.Gln247Gln), c.463C>T (p.Arg155Cys), c.1013C>T (p.Thr338Met) and c.895G>A (p.Gly299Ser). In family 6, *Ectodysplasin A receptor* (*EDAR*) mutation c.1259G>A (p.Arg420Gln) was identified. *WNT10A* mutation c.354T>G (p.Tyr118*) and c.637G>A (p.Gly213Ser) were identified in family 7. Black arrows point to the probands. Red arrows point to the mutations. “+” indicates that the blood sample is available for genetic analysis in this study. In family 1, the sequence chromatogram showed reverse complemented sequence. The remaining sequence chromatograms showed forward sequence.

**Figure 2:**
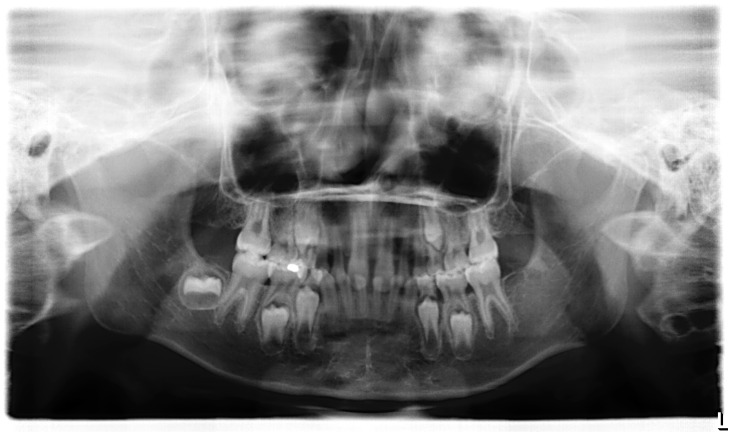
Panoramic radiograph of proband in family 7. He did not have any missing primary teeth congenitally (#51 and #61 had replaced by #11 and #21). However, 15 teeth were missing in the permanent dentition.

The pedigrees are available in Figure 1. The probands of families 2, 3, 5, and 7 presented the triad of hypodontia, hypotrichosis, and hypohidrosis. In family 3, the proband displayed conically-shaped teeth. His grandfather also had these clinical features. Interestingly, the proband of family 7 did not have any missing primary tooth congenitally, while 15 teeth were missing a geneogenous in the permanent dentition (Figure 2). His hair was sparse in his early childhood, according to a report by his mother. As he grew up, his hair became thicker. When he was 11 years old and came to our clinic, we found his hair was not significantly different from that of an average person. The probands of families 1 and 4 manifested hypodontia and hypotrichosis. The proband of family 6 suffered from hypodontia and hypohidrosis, similarly to his mother. The number of the missing teeth in primary and permanent dentition is listed in Table 1. (More clinical information is available in Figure S1 and Table S4.)

### 3.2. Genetic Findings of the *EDA*, *EDAR*, *EDARADD*, and *WNT10A*

We identified eight mutations in seven families (Figure 1; Table 1). Two of the eight mutations were novel mutations. The two novel mutations were not carried by healthy volunteers and were not reported by ExAC, 1000 Genomes, PubMed, or the Human Gene Mutation Database (HGMD) public version (accession date: 3 August 2016) [23]. Families 1–5 harbored heterozygous mutations in *EDA*. In family 6, we identified one *EDAR* mutation. In family 7, two *WNT10A* mutations and two *EDAR* single-nucleotide polymorphisms (SNPs) were found.

#### 3.2.1. Family 1

**Figure 3:**
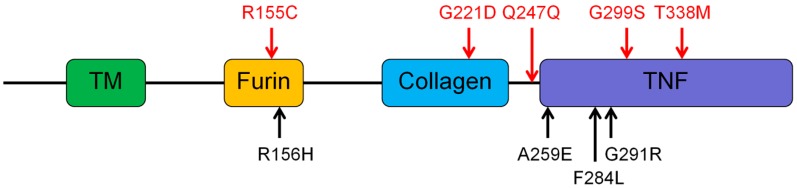
Domains of *Ectodysplasin A* and mutation distribution. Mutations in red are those identified in this study. Mutations in black are those identified in our previous study [5]. Collagen: collagen-like domain; Furin: furin sites; TNF: tumor necrosis factor domain.

**Figure 4:**
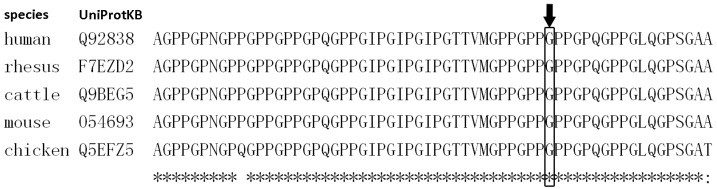
A cross-species alignment of the amino acid sequence of *EDA* indicated that p.Gly221 (in the box) was conserved.

A novel mutation, c.662G>A (p.Gly221Asp) in *EDA* gene, is identified in family 1. It was located at the collagen-like domain (Figure 3), and led to the replacement of glycine by aspartic acid at amino acid residue 221. A cross-species alignment of protein sequences showed that p.Gly221 was evolutionarily conserved (Figure 4). SIFT, PolyPhen2, Mutation Taster, FATHMM, and PROVEAN predicted the mutational effect to be damaging, probably damaging, disease-causing, damaging and deleterious, respectively.

#### 3.2.2. Families 2–6

The four *EDA* mutations identified in families 2–5 were c. 741G>A (p.Gln247Gln, may affect the splice site), c.463C>T (p.Arg155Cys), c.1013C>T (p.Thr338Met), and c.895G>A (p.Gly299Ser), respectively. In family 6, we found *EDAR* c.1259G>A (p.Arg420Gln) mutation in both the genomes of the patient and of his mother. All of the five mutations had been reported by other researchers [9,24,25,26]. The pathogenicity of the mutations is well established.

#### 3.2.3. Family 7

**Figure 5:**
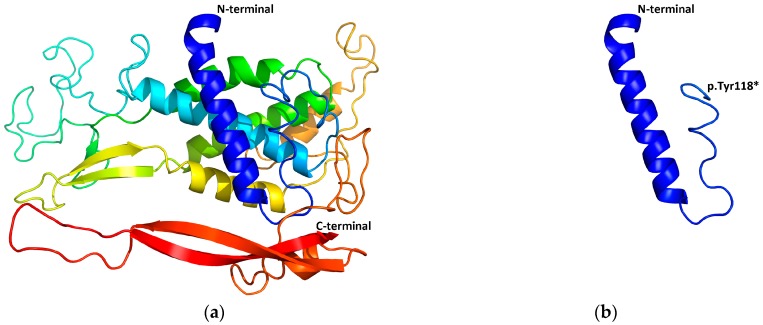
The structure of protein Wnt-8 (PDB ID 4F0A) was used as a homology model to perform structural modeling of the (**a**) wild-type Wnt-10a and (**b**) p.Tyr118* mutated Wnt-10a. The mutated Wnt-10a protein was unlikely to have any function with the majority of the protein structure lost.

Identified in family 7, c.354T>G (p.Tyr118*) mutation in the *WNT10A* gene was carried by the patient and his father, while c.637G>A (p.Gly213Ser) mutation was shared by the patient and his mother. The novel c.354T>G mutation truncated 300 amino acids from the C terminus of the Wnt-10a protein by inducing a termination codon at amino acid position 118. The mutated protein was predicted to undergo nonsense-mediated mRNA decay (NMD) by Mutation Taster [17]. Even if it was not decayed by NMD, the mutated Wnt-10a protein was unlikely to have any function with the majority of the protein structure lost (Figure 5). c.637G>A mutation has been reported to cause oligodontia with minor signs of ectodermal dysplasia in autosomal recessive inheritance [27]. This is in accordance with our case.

**Figure 6:**
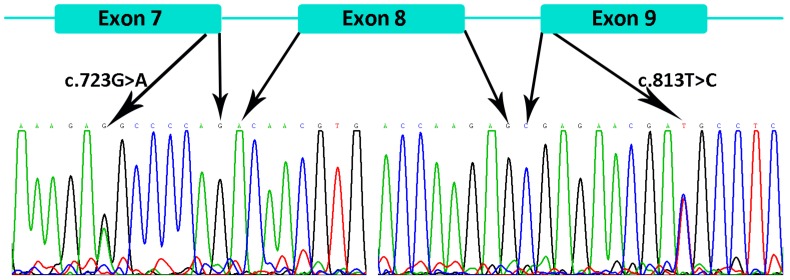
Splicing analysis of *EDAR* gene with total RNA from the patient in family 7. Exons 7–9 of *EDAR* gene and sequencing results are showed. The sequencing results indicated normal splicing of *EDAR* gene with the presence of c.723G>A and c.813T>C variants.

There were two more variants, c.723G>A (p.Glu241Glu) and c.813T>C (p.Asp271Asp) in the *EDAR* gene, identified in the patient of family 7. Mutation Taster predicted the variants were disease causing mutations for they may change the splice site of *EDAR*. However, we considered them as SNPs for the following three reasons. Firstly, splicing analysis of the patient’s total RNA showed normal splicing of *EDAR* (Figure 6). Secondly, these two variants are on the same chromosome that was inherited from his mother, who showed no signs of HED. Thirdly, the homozygous individuals were recorded in 1000 Genomes and ExAC database.

#### 3.2.4. Detection of the *EDAR* Variant rs3827760 (c.1109T>C, p.Val370Ala)

*EDAR* gene of probands in families 6 and 7 was sequenced. Both probands carried a heterozygous C allele of SNP rs3827760.

## 4. Discussion

Although Cluzeau et al. reported 10 HED patients harboring *WNT10A* mutations [12], the disease-causing relationship between *WNT10A* mutations and HED was not confirmed by other scientists. Based on the information from OMIM (National Center for Biotechnology Information, Bethesda, MD, USA) and the latest update on ectodermal dysplasia clinical classification [3], *WNT10A* is not recommended as a candidate gene for HED. In this study, we reported a HED patient caused by compound heterozygous mutations in *WNT10A*. This is new evidence ascertaining *WNT10A* as a candidate gene for HED. With the newly-identified *WNT10A*-related HED patient in our study, we think this is a solid fact that *WNT10A* is a candidate gene for HED. We suggest *WNT10A* to be routinely tested in *EDA*-negative HED patients.

*WNT10A* mutation was reported to cause odonto-onycho-dermal dysplasia (OODD), a form of ED characterized by severe oligodontia, nail dystrophy, palmoplantar hyperkeratosis, and hyperhidrosis [28]. Schopf-Schulz-Passarge syndrome (SSPS) is a form of ED similar to OODD but distinguished by eyelid cysts [29]. Bohring et al. reported *WNT10A* mutations can cause not only OODD, but also SSPS and non-syndromic oligodontia [30]. Variability of phenotypes was shown to be inter- and intra-family [31,32]. This could be partly explained by the effect of some genetic variants. The SNP rs3827760 (c.1109T>C, p.Val370Ala) has been associated with increased hair thickness [33]. In this study, we sequenced the *EDAR* gene of the probands in families 6 and 7, and both of them carried the SNP. Their phenotype of hair was consistent with the genotype. Lately, OODD and SSPS have been proven to be the same disease with variable symptoms caused by *WNT10A* mutations [3,31,32]. The clinical feature of HED is milder than OODD but more serious than oligodontia. We consider OODD, HED and oligodontia represented the full clinical manifestations of *WNT10A*-related ED. It is not rare that mutations of the same gene lead to different phenotypes. For example, mutations in the *EDA* gene are the cause of some XLHED and non-syndromic oligodontia in patients, and the two diseases are considered to be the same disease with different degrees of severity [5,34].

In family 7, we identified a novel mutation c.354T>G (p.Tyr118*) and a known mutation c.637G>A (p.Gly213Ser) in *WNT10A* gene. The novel mutation c.354T>G was predicted to be decayed by NMD or truncate a major part of the protein. It is a loss-of-function mutation [35,36]. Interestingly, the most prevalent mutation c.321C>A (p.Cys107*) and c.682T>A (p.Phe228Ile) did not show up in our study. By reviewing the literature, we found that the two prevalent mutations were frequently reported in European, but not in Asian, subjects [30,37,38,39,40]. Population frequency from the ExAC database also showed that the two mutations are most popular in European but are not carried by Asian subjects [41]. In contrast, although c.637G>A (p.Gly213Ser) was first reported in Europe, it is far more prevalent in Asia [41]. This information should be noted in genetic counseling and prenatal diagnosis. Surprisingly, the ExAC database recorded four c.637G>A (p.Gly213Ser) homozygotes in Asian and 14 c.682T>A (p.Phe228Ile) homozygotes in European subjects. We cannot inquire whether the 18 homozygotes have ectodermal defects. We consider ExAC is a useful database for disease studies, and it will be better if medical records of the subjects are available.

The novel *EDA* mutation, c.662G>A (p.Gly221Asp), is located in the collagen-like domain (Collagen) of Ectodysplasin A protein (Figure 3). Collagen functioned as a connector for the trimerization of Ectodysplasin A [42]. Schneider et al. conducted functional analyses of collagen, and revealed that point mutations in collagen can disrupt the trimerization of Ectodysplasin A [42]. Collagen is composed of 19 repeating peptide triplets of glycine-X-Y with a single interruption [42,43]. Intriguingly, the disease-causing mutations in Collagen have a strong bias to glycine. Twelve (86%) of the 14 mutations in Collagen are mutated at glycine [44]. This phenomenon highlights the important role of glycine in maintaining the structure and function of Collagen. As c.662G>A mutation leads to a glycine being replaced by an aspartic acid in Collagen, the mutated protein probably cannot accomplish trimerization.

In a previous study, we reported four HED patients harbored *EDA* mutations [5]. With the new data in this study, *EDA* mutations were detected in nine of 11 patients (82%). This is in accordance with other studies, which showed a detection rate of 57%–88% [42,45,46,47]. We found mutations in only one patient for *WNT10A* and *EDAR* genes. This is not large enough to give a prevalence. Other studies showed that *WNT10A* or *EDAR* was mutated in 9%–16% of HED patients [12,46,47]. Reviewing the distribution of *EDA* mutations in our cohort, we found that all of the mutations were located in furin sites, Collagen and tumor necrosis factor (TNF) domains, except c.741G>A (p.Gln247Gln) (Figure 3). Mutation c.741G>A may also affect the functional domain because it is located before the TNF domain and predicted to alter the splicing of *EDA*.

ED is a group of diseases with great heterogeneity. To date, only 75 EDs are possible to connect a specific gene or chromosomal region [3]. The genetic diagnoses of the remaining 88 EDs are not possible to obtain. This puts us at a disadvantage because we may not treat the patient effectively if we do not know the genetic defect and the mechanism of the disease. The USA, Europe, and China have launched a precision medicine initiative [48,49]. Our study is an effort to achieve precise diagnosis and eventually to practice precision medicine.

## 5. Conclusions

We conducted a genetic study in seven HED families. Two novel and six known mutations were identified in the *EDA*, *EDAR,* and *WNT10A* genes. Bioinformatics analysis, structural modeling, or splicing analysis proved the pathogenicity of the mutations. This study revealed the genetic basis of seven HED patients and expanded the mutational spectrum. New evidence ascertaining *WNT10A* as a candidate gene for HED was provided.

## Figures and Tables

**Table 1:** Summary of clinical data and mutations in *EDA*, *EDAR,* and *WNT10A* genes in family 1–7.

Family	Patient	Age and Gender	Gene Involved	Nucleotide Change	Amino Acid Change	Domain	Mode of Inheritance	*n* Missing Primary Teeth	*n* Missing Permanent Teeth ^†^
1	II:1	4y, M	*EDA*	**c.662G>A**	**p.Gly221Asp**	Collagen	X-linked	18	ND
2	II:2	8y, M	*EDA*	c. 741G>A	p.Gln247Gln		X-linked	18	26
3	III:1	6y, M	*EDA*	c.463C>T	p.Arg155Cys	Furin	X-linked	5	19
4	III:1	11y, M	*EDA*	c.1013C>T	p.Thr338Met	TNF	X-linked	ND	14
5	II:1	7y, M	*EDA*	c.895G>A	p.Gly299Ser	TNF	X-linked	20	ND
6	II:2	8y, M	*EDAR*	c.1259G>A	p.Arg420Gln	DD	AD	ND	7
	I:2	28y, F	*EDAR*	c.1259G>A	p.Arg420Gln	DD	AD	ND	4
7	II:1	11y, M	*WNT10A*	**c.354T>G**	p.Tyr118*		AR	0	15
			*WNT10A*	c.637G>A	p.Gly213Ser				

Notes: **^†^** Excluding the third molars; bold type indicates novel mutation; y: year; M: male; F: female; Collagen: collagen-like domain; Furin: furin sites; TNF: tumor necrosis factor (TNF) domain; DD: death-like domain; AD: autosomal dominance; AR: autosomal recessive; ND: not defined.

## Supplementary Materials

The following are available online at www.mdpi.com/2073-4425/7/9/65/s1. Table S1: Primers amplifying coding exons and flanking intronic sequences of *EDAR*; Table S2: Primers amplifying coding exons and flanking intronic sequences of *EDARADD*; Table S3: Primers amplifying coding exons and flanking intronic sequences of *WNT10A*; Figure S1: Panoramic radiograph of proband in family 2 (a); 3 (b); 4 (c) and 6 (d); Table S4: Detail clinical data of the patients.

## References

[1] Visinoni A.F., Lisboa-Costa T., Pagnan N.A., Chautard-Freire-Maia E.A. (2009). Ectodermal dysplasias: clinical and molecular review. Am. J. Med. Genet. A.

[2] Freire-Maia N. (1971). Ectodermal dysplasias. Hum. Hered..

[3] Pagnan N.A., Visinoni A.F. (2014). Update on ectodermal dysplasias clinical classification. Am. J. Med. Genet. A.

[4] Mikkola M.L. (2009). Molecular aspects of hypohidrotic ectodermal dysplasia. Am. J. Med. Genet. A.

[5] Zeng B., Lu H., Xiao X., Zhou L., Lu J., Zhu L., Yu D., Zhao W. (2015). Novel *EDA* mutation in X-linked hypohidrotic ectodermal dysplasia and genotype-phenotype correlation. Oral Dis..

[6] Deshmukh S., Prashanth S. (2012). Ectodermal dysplasia: a genetic review. Int. J. Clin. Pediatr. Dent..

[7] Kere J., Srivastava A.K., Montonen O., Zonana J., Thomas N., Ferguson B., Munoz F., Morgan D., Clarke A., Baybayan P. (1996). X-linked anhidrotic (hypohidrotic) ectodermal dysplasia is caused by mutation in a novel transmembrane protein. Nat. Genet..

[8] Headon D.J., Emmal S.A., Ferguson B.M., Tucker A.S., Justice M.J., Sharpe P.T., Zonana J., Overbeek P.A. (2001). Gene defect in ectodermal dysplasia implicates a death domain adapter in development. Nature.

[9] Monreal A.W., Ferguson B.M., Headon D.J., Street S.L., Overbeek P.A., Zonana J. (1999). Mutations in the human homologue of mouse dl cause autosomal recessive and dominant hypohidrotic ectodermal dysplasia. Nat. Genet..

[10] Wisniewski S.A., Trzeciak W.H. (2012). A new mutation resulting in the truncation of the *TRAF6*-interacting domain of *XEDAR*: a possible novel cause of hypohidrotic ectodermal dysplasia. J. Med. Genet..

[11] Wisniewski S.A., Trzeciak W.H. (2012). A rare heterozygous *TRAF6* variant is associated with hypohidrotic ectodermal dysplasia. Br. J. Dermatol..

[12] Cluzeau C., Hadj-Rabia S., Jambou M., Mansour S., Guigue P., Masmoudi S., Bal E., Chassaing N., Vincent M.C., Viot G. (2011). Only four genes (*EDA1*, *EDAR*, *EDARADD*, and *WNT10A*) account for 90% of hypohidrotic/anhidrotic ectodermal dysplasia cases. Hum. Mutat..

[13] Zeng B., Li R., Hu Y., Hu B., Zhao Q., Liu H., Yuan P., Wang Y. (2016). A novel mutation and a known mutation in the *CLCN7* gene associated with relatively stable infantile malignant osteopetrosis in a Chinese patient. Gene.

[14] Lu H., Zeng B., Yu D., Jing X., Hu B., Zhao W., Wang Y. (2015). Complex dental anomalies in a belatedly diagnosed cleidocranial dysplasia patient. Imaging Sci. Dent..

[15] Larkin M.A., Blackshields G., Brown N.P., Chenna R., McGettigan P.A., McWilliam H., Valentin F., Wallace I.M., Wilm A., Lopez R. (2007). Clustal W and Clustal X version 2.0. Bioinformatics.

[16] Ng P.C., Henikoff S. (2001). Predicting deleterious amino acid substitutions. Genome Res..

[17] Schwarz J.M., Cooper D.N., Schuelke M., Seelow D. (2014). MutationTaster2: mutation prediction for the deep-sequencing age. Nat. Methods.

[18] Adzhubei I.A., Schmidt S., Peshkin L., Ramensky V.E., Gerasimova A., Bork P., Kondrashov A.S., Sunyaev S.R. (2010). A method and server for predicting damaging missense mutations. Nat. Methods.

[19] Shihab H.A., Gough J., Cooper D.N., Stenson P.D., Barker G.L., Edwards K.J., Day I.N., Gaunt T.R. (2013). Predicting the functional, molecular, and phenotypic consequences of amino acid substitutions using hidden Markov models. Hum. Mutat..

[20] Choi Y., Sims G.E., Murphy S., Miller J.R., Chan A.P. (2012). Predicting the functional effect of amino acid substitutions and indels. PLoS ONE.

[21] Choi Y. A fast computation of pairwise sequence alignment scores between a protein and a set of single-locus variants of another protein. Proceeding of the ACM Conference on Bioinformatics, Computational Biology and Biomedicine (BCB '12).

[22] Guex N., Peitsch M.C. (1997). SWISS-MODEL and the Swiss-PdbViewer: an environment for comparative protein modeling. Electrophoresis.

[23] Richards S., Aziz N., Bale S., Bick D., Das S., Gastier-Foster J., Grody W.W., Hegde M., Lyon E., Spector E. (2015). Standards and guidelines for the interpretation of sequence variants: a joint consensus recommendation of the American College of Medical Genetics and Genomics and the Association for Molecular Pathology. Genet. Med..

[24] Conte C., Gambardella S., Bulli C., Rinaldi F., Di Marino D., Falconi M., Bramanti P., Desideri A., Novelli G. (2008). Screening of *EDA1* gene in X-linked anhidrotic ectodermal dysplasia using DHPLC: identification of 14 novel mutations in Italian patients. Genet. Test..

[25] Monreal A.W., Zonana J., Ferguson B. (1998). Identification of a new splice form of the *EDA1* gene permits detection of nearly all X-linked hypohidrotic ectodermal dysplasia mutations. Am. J. Hum. Genet..

[26] Li S., Li J., Cheng J., Zhou B., Tong X., Dong X., Wang Z., Hu Q., Chen M., Hua Z.C. (2008). Non-syndromic tooth agenesis in two Chinese families associated with novel missense mutations in the TNF domain of EDA (ectodysplasin A). PLoS ONE.

[27] Plaisancie J., Bailleul-Forestier I., Gaston V., Vaysse F., Lacombe D., Holder-Espinasse M., Abramowicz M., Coubes C., Plessis G., Faivre L. (2013). Mutations in *WNT10A* are frequently involved in oligodontia associated with minor signs of ectodermal dysplasia. Am. J. Med. Genet. A.

[28] Adaimy L., Chouery E., Megarbane H., Mroueh S., Delague V., Nicolas E., Belguith H., de Mazancourt P., Megarbane A. (2007). Mutation in *WNT10A* is associated with an autosomal recessive ectodermal dysplasia: The odonto-onycho-dermal dysplasia. Am. J. Hum. Genet..

[29] Schopf E., Schulz H.J., Passarge E. (1971). Syndrome of cystic eyelids, palmo-plantar keratosis, hypodontia and hypotrichosis as a possible autosomal recessive trait. Birth Defects Orig. Artic. Ser..

[30] Bohring A., Stamm T., Spaich C., Haase C., Spree K., Hehr U., Hoffmann M., Ledig S., Sel S., Wieacker P. (2009). *WNT10A* mutations are a frequent cause of a broad spectrum of ectodermal dysplasias with sex-biased manifestation pattern in heterozygotes. Am. J. Hum. Genet..

[31] Vink C.P., Ockeloen C.W., Ten K.S., Koolen D.A., Ploos V.A.J., Kuijpers-Jagtman A.M., van Heumen C.C., Kleefstra T., Carels C.E. (2014). Variability in dentofacial phenotypes in four families with *WNT10A* mutations. Eur. J. Hum. Genet..

[32] Wedgeworth E.K., Nagy N., White J.M., Pembroke A.C., McGrath J.A. (2011). Intra-familial variability of ectodermal defects associated with *WNT10A* mutations. Acta Derm. Venereol..

[33] Mou C., Thomason H.A., Willan P.M., Clowes C., Harris W.E., Drew C.F., Dixon J., Dixon M.J., Headon D.J. (2008). Enhanced *ectodysplasin-A receptor* (*EDAR*) signaling alters multiple fiber characteristics to produce the East Asian hair form. Hum. Mutat..

[34] Gaczkowska A., Abdalla E.M., Dowidar K.M., Elhady G.M., Jagodzinski P.P., Mostowska A. (2016). De novo *EDA* mutations: Variable expression in two Egyptian families. Arch. Oral Biol..

[35] Rivas M.A., Pirinen M., Conrad D.F., Lek M., Tsang E.K., Karczewski K.J., Maller J.B., Kukurba K.R., DeLuca D.S., Fromer M. (2015). Human genomics. Effect of predicted protein-truncating genetic variants on the human transcriptome. Science.

[36] Chaudhary A.K., Sankar V.H., Bashyam M.D. (2016). A novel large deletion that encompasses *EDA* and the downstream gene *AWAT2* causes X-linked hypohidrotic/anhidrotic ectodermal dysplasia. J. Dermatol. Sci..

[37] Van den Boogaard M.J., Creton M., Bronkhorst Y., van der Hout A., Hennekam E., Lindhout D., Cune M., Ploos V.A.H. (2012). Mutations in *WNT10A* are present in more than half of isolated hypodontia cases. J. Med. Genet..

[38] Mostowska A., Biedziak B., Zadurska M., Dunin-Wilczynska I., Lianeri M., Jagodzinski P.P. (2013). Nucleotide variants of genes encoding components of the Wnt signalling pathway and the risk of non-syndromic tooth agenesis. Clin. Genet..

[39] Arzoo P.S., Klar J., Bergendal B., Norderyd J., Dahl N. (2014). *WNT10A* mutations account for (1/4) of population-based isolated oligodontia and show phenotypic correlations. Am. J. Med. Genet. A.

[40] Mues G., Bonds J., Xiang L., Vieira A.R., Seymen F., Klein O., D'Souza R.N. (2014). The *WNT10A* gene in ectodermal dysplasias and selective tooth agenesis. Am. J. Med. Genet. A.

[41] Lek M., Karczewski K.J., Minikel E.V., Samocha K.E., Banks E., Fennell T., O'Donnell-Luria A.H., Ware J.S., Hill A.J., Cummings B.B. (2016). Analysis of protein-coding genetic variation in 60,706 humans. Nature.

[42] Schneider P., Street S.L., Gaide O., Hertig S., Tardivel A., Tschopp J., Runkel L., Alevizopoulos K., Ferguson B.M., Zonana J. (2001). Mutations leading to X-linked hypohidrotic ectodermal dysplasia affect three major functional domains in the tumor necrosis factor family member ectodysplasin-A. J. Biol. Chem..

[43] Li D., Xu R., Huang F., Wang B., Tao Y., Jiang Z., Li H., Yao J., Xu P., Wu X. (2015). A novel missense mutation in collagenous domain of *EDA* gene in a Chinese family with X-linked hypohidrotic ectodermal dysplasia. J. Genet..

[44] HGMD public version. http://www.hgmd.cf.ac.uk/ac/all.php.

[45] Vincent M.C., Biancalana V., Ginisty D., Mandel J.L., Calvas P. (2001). Mutational spectrum of the *ED1* gene in X-linked hypohidrotic ectodermal dysplasia. Eur. J. Hum. Genet..

[46] Van der Hout A.H., Oudesluijs G.G., Venema A., Verheij J.B., Mol B.G., Rump P., Brunner H.G., Vos Y.J., van Essen A.J. (2008). Mutation screening of the Ectodysplasin-A receptor gene *EDAR* in hypohidrotic ectodermal dysplasia. Eur. J. Hum. Genet..

[47] Wohlfart S., Hammersen J., Schneider H. (2016). Mutational spectrum in 101 patients with hypohidrotic ectodermal dysplasia and breakpoint mapping in independent cases of rare genomic rearrangements. J. Hum. Genet..

[48] Collins F.S., Varmus H. (2015). A new initiative on precision medicine. N. Engl. J. Med..

[49] Kinkorova J. (2014). [Horizon 2020, new EU Framework programme for research and innovation, 2014-2020]. Cas. Lek. Cesk..

