# Supplementary material for: Eight Mutations of Three Genes (EDA, EDAR, and WNT10A) Identified in Seven Hypohidrotic Ectodermal Dysplasia Patients
Source: Genes (Basel). 2016 Sep 19;7(9):65. doi: 10.3390/genes7090065 (PMC5042395; doi:10.3390/genes7090065)
Supplement: Supplementary file 1 [file genes-07-00065-s001.docx]

Supplementary Materials: Eight Mutations of Three Genes (*EDA*, *EDAR* and *WNT10A*) Identified in Seven Hypohidrotic Ectodermal Dysplasia Patients

Binghui Zeng, Xue Xiao, Sijie Li, Hui Lu, Jiaxuan Lu, Ling Zhu, Dongsheng Yu and Wei Zhao

**Table S1.** Primers amplifying coding exons and flanking intronic sequences of *EDAR*.

| **Exon** | **Forward (5′–3′)** | **Reverse (5′–3′)** |
| --- | --- | --- |
| **2** | GCTTCATGTCTGCTTTTCTTTTA | GTAACTTCAGCCTGGGGCAGAGT |
| **3** | CAGCAGAGCCCCCACCGCAGGGC | GCCATCAATCTCAACGTCCAGGGAGC |
| **4** | CCTGGGGGGTTTTGCAGTTAG | CCTGGATGCTGCTCCTTGTGG |
| **5** | CTGAAACCCCAAGGAAACTGAG | GCACTAGAACCTCAGCCTCTGAC |
| **6** | GAAATACAAATATTTAGCTCCCCTC | GATCAACTCTGATTGCAGATATAATG |
| **7-9** | AGGATTTGGTTCTCAGTCTAAGCA | CTCCTCACACCACCTGACCCC |
| **10** | CTCGTCTCATGTCCCCTGGTCC | GACTATCTTGCTGTGAACTTGTCACTG |
| **11** | CCCAAAAATGTTTGAATAGGAAAAC | CAGCGGCCATTCTGACCAAAGTG |
| **12** | CATTTAAATAATTTTTCAGTGTTGACC | GGCTCCTTGAACATCCTAAGGCATAC |

**Table S2.** Primers amplifying coding exons and flanking intronic sequences of *EDARADD*.

| **Exon** | **Forward (5′–3′)** | **Reverse (5′–3′)** |
| --- | --- | --- |
| **1a** | GAAGGCAGACCAAGAGGAAGTTT | GCTCTAAATAATCTTTCTCTGCC |
| **1b** | GCGAAGGGGCTTTGCTAATTGC | GTCAGCAGCCGCTCGCGGGGC |
| **2** | GAGTAAGGTTTTCTTCAGCCTAAG | GAAAAATCTTACTTTGGGGTTTC |
| **3** | GGATAAGGCCAGTTGATAAGTTTG | GACAATTATGTCACAAGTCACGAGC |
| **4** | GAGCAGAGTTTGGAGAGGAATTTG | GAGACCATTTGCAAACCCTGTTT |
| **5** | GCTGAGTTCACCTCCCATTAAAATG | GTATTTTTGGTAGAGACGGGGTTTT |
| **6** | CGAGCATTCTGAAATAGTCTTCCA | AACTGCCAGCTTTGGGGAAAAC |

Notes: Exon 1a is Exon 1 of RefSeq [NM_145861](http://www.ncbi.nlm.nih.gov/nuccore/NM_145861), and Exon 1b is Exon 1 of RefSeq [NM_080738](http://www.ncbi.nlm.nih.gov/nuccore/NM_080738).

| 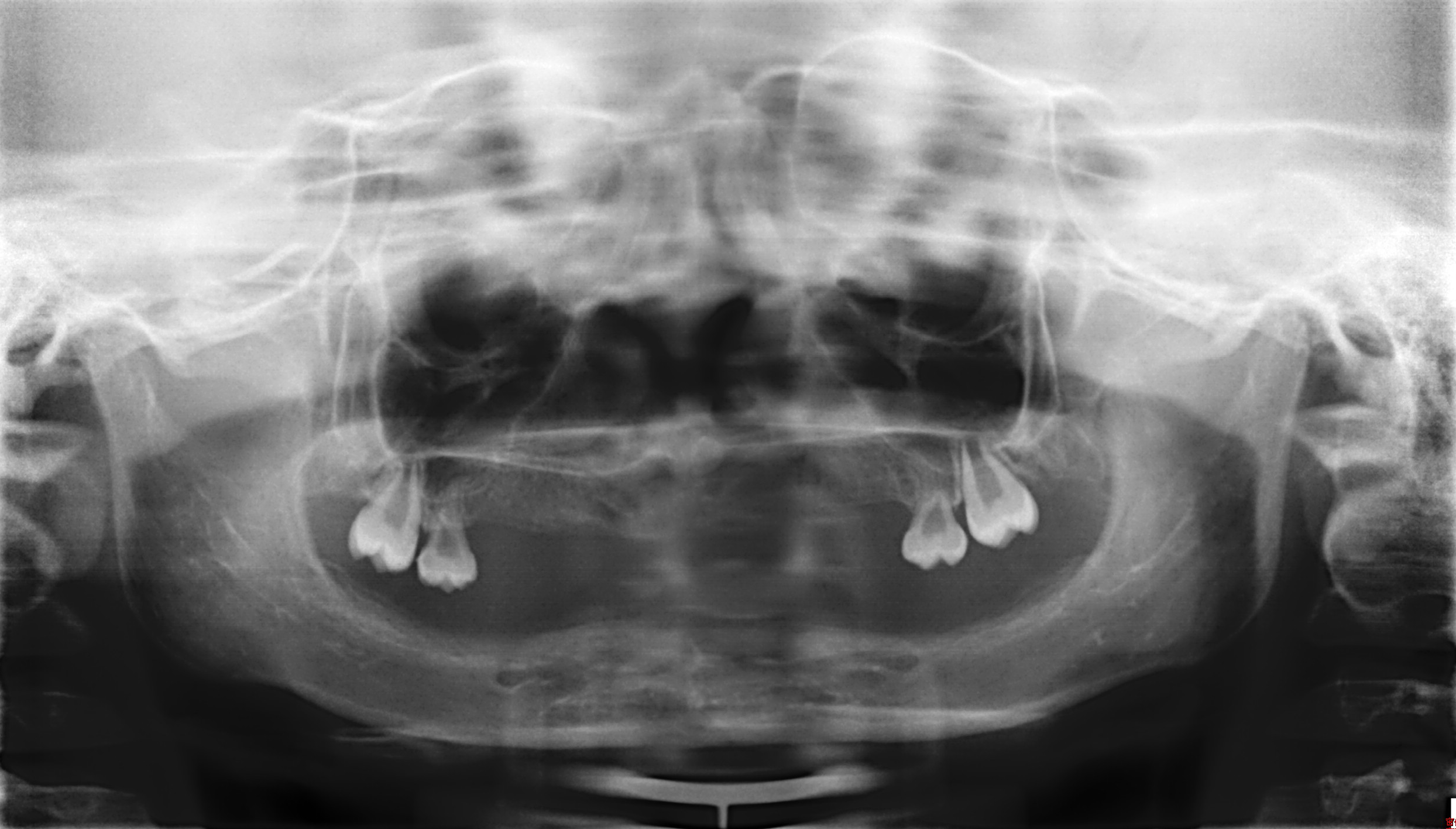 |
| --- |
| (**a**) |
| 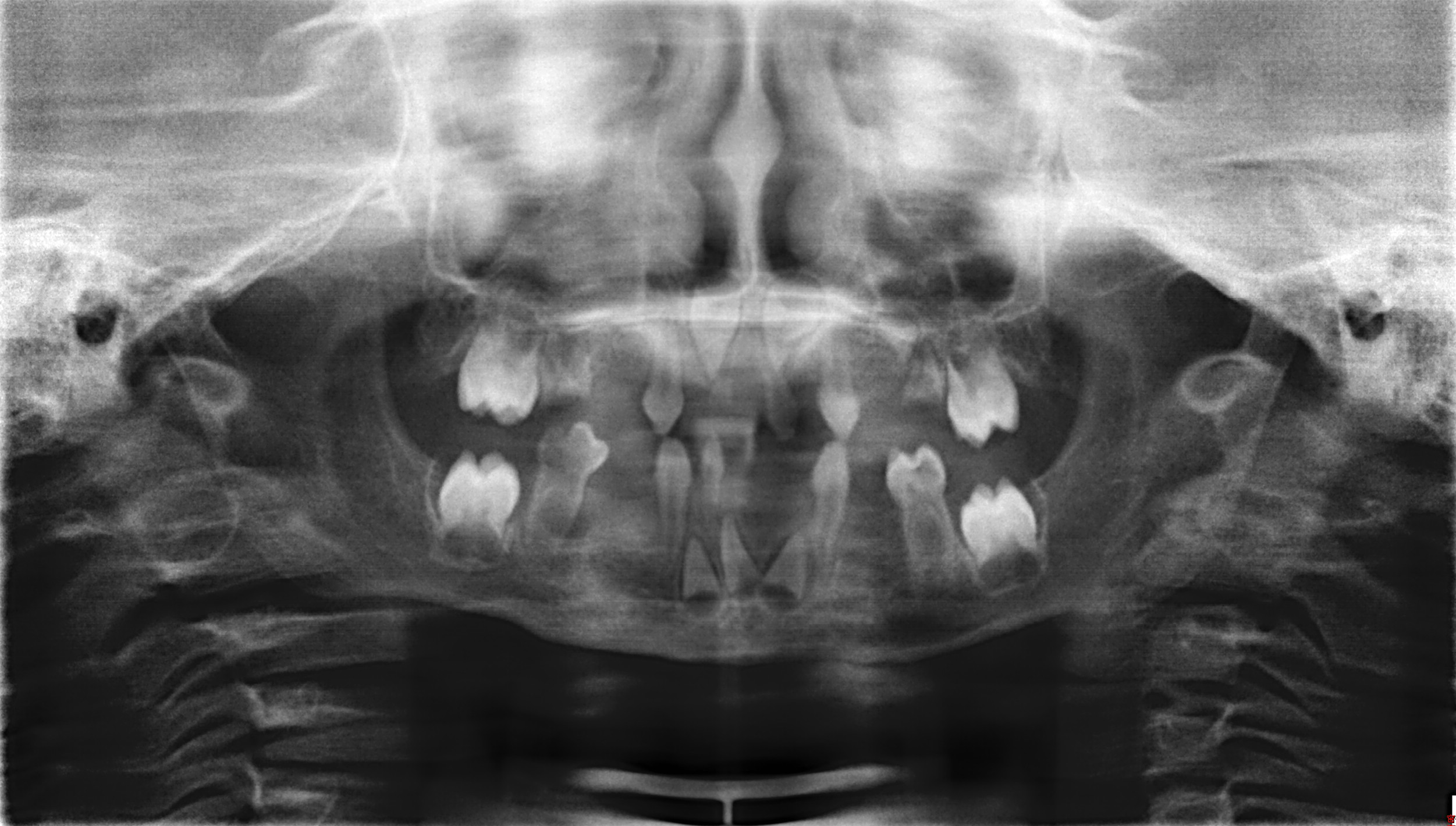 |
| (**b**) |
| 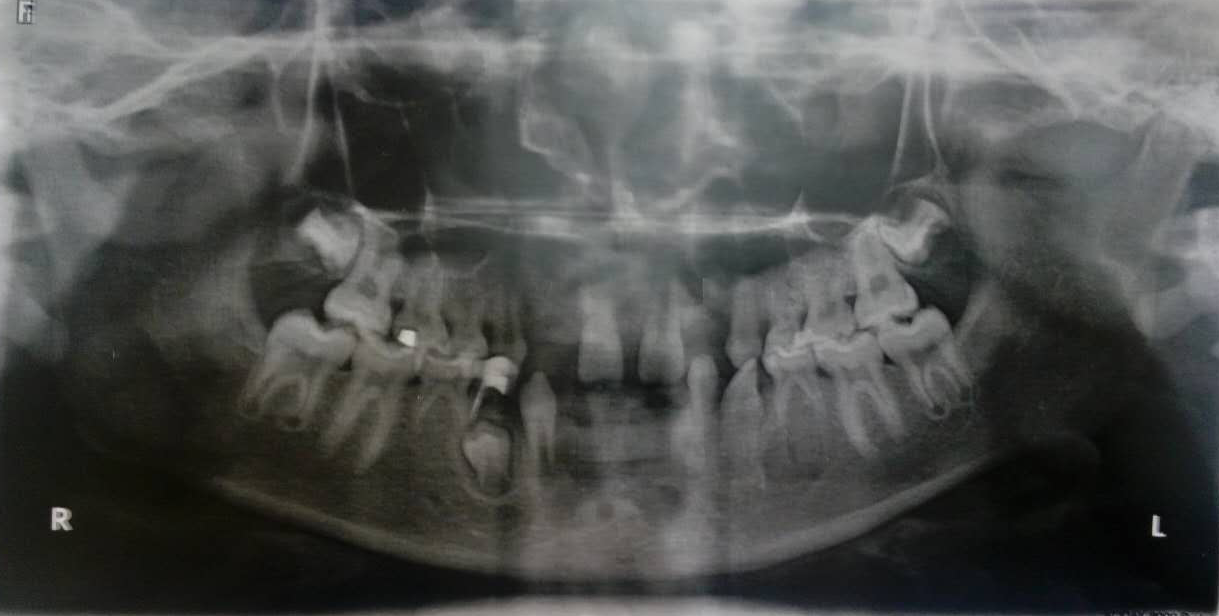 |
| (**c**) |
| 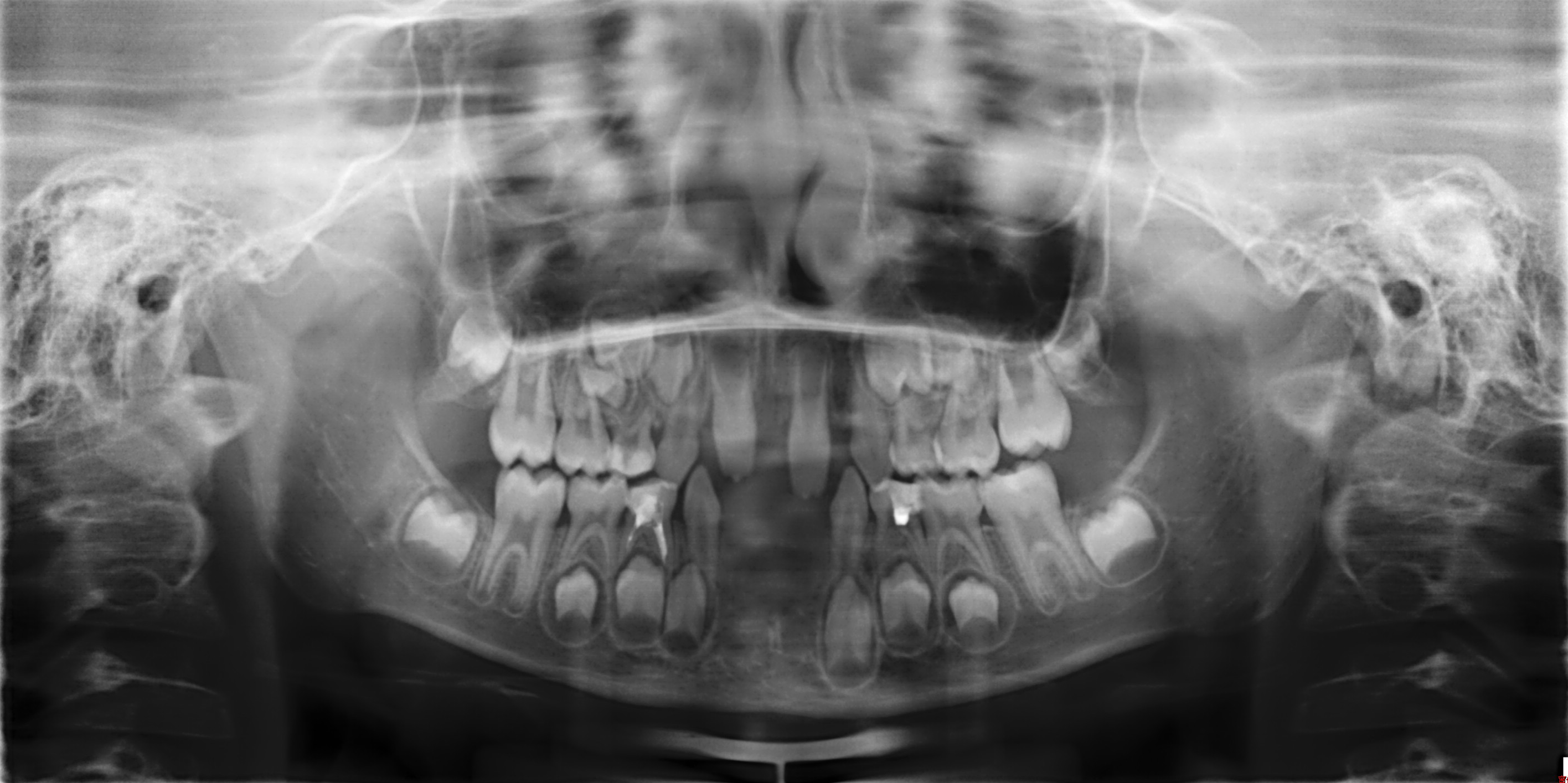 |
| (**d**) |

**Figure S1.** Panoramic radiograph of proband in family 2 (**a**), 3 (**b**), 4 (**c**) and 6 (**d**). (**a**) In family 2, the proband had 18 primary and 26 permanent teeth missing; (**b**) In family 3, the proband had 5 primary and 19 permanent teeth missing. The incisors and primary incisors were in conic shape; (**c**) In family 4, the proband had 14 permanent teeth missing; (**d**) In family 6, the proband had 7 permanent teeth missing. The number of missing primary teeth of the patients of families 4 and 6 could not be defined because some of the primary teeth should be replaced by permanent teeth at 11 years old or 8 years old. Panoramic radiograph of proband in family 1 and 5 could not be obtained because the patients were unwilling to take the examination.

**Table S3.** Primers amplifying coding exons and flanking intronic sequences of *WNT10A*.

| **Exon** | **Forward (5′–3′)** | **Reverse (5′–3′)** |
| --- | --- | --- |
| **1** | GCTGCCCCATGGAGCGGGGAG | GCCCATATCCTGTCACCAGTCCAAC |
| **2** | CAAGGAGAGAGGAATGTGTAGGAG | GCCTGAGATCAGAAAGAGGAAGG |
| **3** | CAGAAGAACTGGCTTCTGGCGTG | GCCTAGTCCAGTGCCTGGTGCAC |
| **4** | CTGACTGCCTGGTTGTGGGACC | GACTGTCCGGCGATCACAGACC |

**Table S4.** Detail clinical data of the patients.

| **Family** | **Patient** | **Age and Gender** | **Number of Missing Primary Teeth** | **Number of Missing Permanent Teeth *** | **Crown Shape** | **Sweat Glands** | **Hair** | **Nails** | **Other Signs** |
| --- | --- | --- | --- | --- | --- | --- | --- | --- | --- |
| **1** | II:1 | 4y, M | 18 | ND | Normal | Normal | Hypotrichosis | Normal |  |
| **2** | II:2 | 8y, M | 18 | 26 | Normal | Hypohidrosis | Hypotrichosis | Normal | Dry skin, intolerance to heat |
| **3** | III:1 | 6y, M | 5 | 19 | Conic | Hypohidrosis | Hypotrichosis | Normal |  |
| **4** | III:1 | 11y, M | ND | 14 | Normal | Normal | Hypotrichosis | Normal |  |
| **5** | II:1 | 7y, M | 20 | ND | Normal | Hypohidrosis | Hypotrichosis | Normal |  |
| **6** | II:2 | 8y, M | ND | 7 | Normal | Hypohidrosis | Normal | Normal | Dry skin |
|  | I:2 | 28y, F | ND | 4 | Normal | Hypohidrosis | Normal | Normal |  |
| **7** | II:1 | 11y, M | 0 | 15 | Normal | Hypohidrosis | Sparse | Normal | Eczema |

Notes: * Excluding the third molars; y: year; M: male; F: female; ND: not defined.
